# Evidentiary Standards for Newly Approved Antibiotics for Uncomplicated Urinary Tract Infections

**DOI:** 10.3390/antibiotics15030238

**Published:** 2026-02-25

**Authors:** Rosa Rodriguez-Monguio, Enrique Seoane-Vazquez, John H. Powers

**Affiliations:** 1 Department of Clinical Pharmacy, School of Pharmacy, University of California San Francisco, San Francisco, CA 94143, USA; 2 Medication Outcomes Center, University of California San Francisco, San Francisco, CA 94143, USA; 3 Philip R. Lee Institute for Health Policy Studies, University of California San Francisco, San Francisco, CA 94143, USA; 4 Division of Clinical Informatics and Digital Transformation, Department of Medicine, University of California San Francisco, 10 Koret Way, San Francisco, CA 94143, USA; 5 Department of Pharmaceutical Economics and Policy, School of Pharmacy, Chapman University, Irvine, CA 92618, USA; seoanevazquez@chapman.edu; 6 Economic Science Institute, Argyros School of Business and Economics, Chapman University, Orange, CA 92866, USA; 7 Department of Medicine, School of Medicine, George Washington University, Washington, DC 20052, USA; jpowers3@aol.com

**Keywords:** antimicrobial use, food and drug administration, evidentiary standards, antimicrobial resistance, rational use of medicines

## Abstract

**Importance**: Uncomplicated urinary tract infections (uUTIs) are among the most common bacterial infections and are typically treated with existing oral antibiotics. In 2024–2025, the FDA approved two new oral agents, sulopenem etzadroxil/probenecid and gepotidacin, via expedited review pathways, for the treatment of uUTIs. **Objective**: To assess the clinical significance and regulatory evidence supporting FDA approval of sulopenem/probenecid and gepotidacin for uUTI, and to analyze the adherence of pivotal phase III trials to regulatory standards for approval and FDA guidelines. **Design, Setting, and Participants**: Comparative descriptive analysis of publicly available data from phase 3 randomized, double-blind, active-controlled clinical trials submitted to the FDA for approval. Pivotal phase III clinical trial data and FDA integrated reviews, guidance for the industry, and approved drug labels. Adult women with uUTI enrolled in pivotal phase III clinical trials, with subgroup analysis stratified by pathogen susceptibility to comparator antibiotics. **Interventions**: Sulopenem/probenecid was compared to ciprofloxacin and to amoxicillin/clavulanate and gepotidacin was compared to nitrofurantoin. **Main Outcomes and Measures**: Primary endpoints were clinical and microbiological responses assessed in the microbiologic modified intention-to-treat (micro-MITT) subjects. Safety outcomes and FDA regulatory determinations were also assessed. **Results**: Sulopenem/probenecid was inferior to ciprofloxacin and noninferior to amoxicillin/clavulanate in subjects with susceptible pathogens, and superior in subjects with resistant pathogens. Gepotidacin showed noninferiority to nitrofurantoin in one trial and superiority in another. Trials excluded randomized subjects, included post-randomization subgroup analyses, and enrolled control-arm subjects with resistant pathogens. Clinical cure rates were lower than historical comparators. Both new antibiotics had higher adverse event rates than controls. **Conclusions and Relevance**: Pivotal clinical trials for sulopenem/probenecid and gepotidacin for uUTI had significant design limitations and relied on surrogate endpoints of limited clinical interpretability, undermining reliability and clinical relevance. Future antibiotic development for uUTI should prioritize representative populations, standard-of-care comparators, clinically meaningful outcomes and robust, well-controlled trial designs to ensure meaningful clinical evidence of safety and efficacy.

## 1. Introduction

Antibiotics are widely used to treat uncomplicated urinary tract infections (uUTIs), a self-limiting condition with minimal risk of progression to serious infection or sepsis [1,2]. Evidence from placebo-controlled trials indicates that 25% to 42% of patients with uUTIs experience symptomatic resolution and no studies have reported mortality or irreversible morbidity associated with uUTI [1,3]. Nearly 28 years after the approval of levofloxacin in 1996, the FDA has approved two new oral antibiotics for uUTIs: sulopenem etzadroxil/probenecid and gepotidacin. The FDA granted Qualified Infectious Disease Product (QIDP) designation, enabling these drugs to undergo fast-track and priority review [4].

*Sulopenem*/probenecid is a formulation of sulopenem combined with probenecid. Sulopenem is not a first-in-class antibiotic and shares the beta-lactam mechanism of action. Sulopenem/probenecid trials for complicated intra-abdominal infection (cIAI) [5] and complicated urinary tract infection (cUTI) [6] failed to meet their primary endpoints [7]. The first new drug application (NDA) for sulopenem/probenecid was submitted to the FDA in November 2020 for the treatment of uUTI in adult women caused by microorganisms proven or strongly suspected to be non-susceptible to quinolones. In July 2021, the FDA determined that sulopenem/probenecid was inferior to active controls used in the clinical trial and recommended another trial to establish substantial evidence of efficacy [7]. In April 2024, the sponsor company resubmitted the NDA, and on 25 October 2024, the FDA approved sulopenem/probenecid for the treatment of uUTI in adult women with limited or no alternative oral antibacterial treatment options caused by susceptible pathogens [8].

Gepotidacin is a first-in-class triazaacenaphthylene antibacterial drug with a mechanism of action similar to that of fluoroquinolones [9]. It was initially studied for the treatment of skin and skin structure infections [10] and uncomplicated urogenital gonorrhea [11,12]. The NDA for gepotidacin was submitted to the FDA in July 2024 and approved on 17 March 2025 for the treatment of uUTIs caused by susceptible pathogens.

Previous studies have reviewed the efficacy and safety of newly approved antibiotics [13,14,15]. However, no studies have comprehensively examined how regulatory standards and FDA guidelines are applied to determine evidence of safety and efficacy for these antibiotics. This study addresses this knowledge gap by critically evaluating the evidence supporting FDA approval of sulopenem/probenecid and gepotidacin. This study also evaluates adherence to FDA regulations and guidance and implications for clinical practice, patient benefits and safety. Our findings provide critical insights into the evidentiary standards for antibiotic development and highlight considerations to inform evaluation of future anti-infective therapies.

## 2. Results

### 2.1. Evidence of Effectiveness

Sulopenem/probenecid uUTI efficacy data were derived from two noninferiority, phase 3, randomized, double-blind, active-controlled trials (Table 1) [16,17]. The primary endpoint was the composite clinical and microbiological response in the micro-MITTS population. The analysis was conducted in the microbiologic modified intention-to-treat (micro-MITT) population, breaking down the analysis for subjects in the active control-susceptible (micro-MITTS) and resistant bacteria (micro-MITTR) subgroups.

In trial 301, sulopenem/probenecid 1000 mg/1000 mg for five days was statistically inferior to ciprofloxacin 500 mg for three days for the composite outcome in the micro-MITTS population and statistically superior for the micro-MITTR population (Table 2). The FDA concluded that results from trial 301 for uUTI and trials for other indications did not demonstrate effectiveness for uUTI [7].

Trial 310 compared sulopenem/probenecid 1000 mg/1000 mg for five days to amoxicillin–clavulanate potassium (amoxicillin/clavulanate) 1750 mg/250 mg for five days (Table 2). The FDA concluded that trial 310 demonstrated sulopenem/probenecid noninferiority for the micro-MITTS and superiority for the micro-MITTR subgroups [7]. In trial 310, outcomes for the micro-MITTR subgroup favored amoxicillin/clavulanate; however, the sample size included 67 subjects [7].

Gepotidacin uUTI clinical efficacy data were derived from two phase 3, randomized, multicenter, double-blind, active-controlled noninferiority trials comparing five days of twice daily gepotidacin 1500 mg and nitrofurantoin 100 mg (Table 1). The primary endpoint was a composite endpoint assessing clinical and microbiological response. Analyses were conducted in the micro-MITT, micro-MITTS, and micro-MITTR subgroups. Based on interim analyses, the FDA concluded that gepotidacin was noninferior to nitrofurantoin in trial EAGLE2 and superior in trial EAGLE-3 (Table 3) [7].

In trial 310, sulopenem/probenecid micro-MITT analysis included 1071 subjects (54.1% of randomized subjects) and the micro-MITTS analysis included 785 subjects (47.0%) [7]. In trial 310, the micro-MITT analysis included 991 subjects (44.6%) and the micro-MITTS analysis included 922 subjects (41.5%) [7]. Sulopenem/probenecid clinical response was assessed at the end of treatment (Day 5 after randomization), test-of-cure (TOC) (Day 12 ± 1), and follow-up visit (FV) (Day 28 ± 2). In trial 301, the treatment duration was 5 days for sulopenem and 3 days for ciprofloxacin. Thus, TOC took place 7 to 9 days after end of treatment, depending on the study arm. In gepotidacin trials, the planned study visits were on-therapy (Day 2–4), TOC (Day 10–13), and FV (Day 28 ± 3) [18]. However, only outcomes at TOC and FV were presented to the FDA for the gepotidacin approval review.

Gepotidacin trial EAGLE-2 micro-MITT analysis included 766 subjects (50.0% of the randomized subjects) and the micro-MITTS 634 subjects (41.4%) [7]. Trial EAGLE-3 micro-MITT analysis included 655 patients (40.8%), and the micro-MITTS 567 patients (35.3%). The gepotidacin interim analysis was conducted for 541 subjects in the micro-MITTS subgroup. The results of the two gepotidacin clinical trials excluded subjects for whom the response was “unable to determine” [9]. The percentage of patients with outcomes “unable to determine” for microbiological success and clinical success were higher in the gepotidacin study arm than in the active comparator nitrofurantoin [9].

While there were differences in the composite, clinical, and microbiological outcomes in sulopenem/probenecid and gepotidacin clinical trials, the clinical cure rates for the MTTS subgroups were not significantly different between sulopenem/probenecid and gepotidacin and their active controls. Notably, patient-reported clinical response rates were lower than investigator-determined clinical success. In trial 310, for example, patient-reported clinical response in the micro-MITTS subgroup was 77.3% for sulopenem/probenecid and 76.7% for amoxicillin/clavulanate, whereas the investigator-determined clinical success was 87.7% and 87.3%, respectively [7].

### 2.2. Safety Profile

Subjects in the sulopenem/probenecid arm experienced a 21.5% incidence rate of adverse events compared to 12.3% in the amoxicillin/clavulanate and 14.0% in the ciprofloxacin active control arms (Table 4). The difference in the incidence of adverse events was driven by infections, gastro-intestinal, and nervous system events.

Additionally, the incidence rate of serious adverse events, including neurologic adverse events, hypersensitivity reactions, QTc prolongation, and *Clostridioides difficile* infection, leading to discontinuation was higher among subjects in the gepotidacin (5.0%) arm than the active control (1.9%) arm (Table 4) [9]. The FDA required inclusion of warnings in the prescribing information and a medication guide to communicate these risks to healthcare providers and patients [9].

## 3. Discussion

The FDA granted sulopenem/probenecid and gepotidacin the Qualified Infectious Disease Product (QIDP) designation, which enabled both drugs to undergo fast-track and priority review [4]. The QIDP designation is intended for drugs indicated to treat serious or life-threatening infections caused by antibacterial or antifungal resistant pathogens or other qualifying pathogens [4,19,20]. Priority review and fast-track designations are intended to expedite patients’ access to therapies that address serious or life-threatening conditions [19] rather than self-limiting conditions such as uUTI that rarely progress to serious infection. Neither sulopenem/probenecid nor gepotidacin were clinically assessed against any qualifying pathogen that poses a significant public health threat, such as multidrug-resistant Gram-negative bacteria [21]. The sulopenem/probenecid approval label explicitly states that the drug is not indicated for treatment of cUTI [8].

### 3.1. Standards for Substantial Evidence of Efficacy

The FDA guidance for uUTI drug development recommends either two adequate and well-controlled clinical trials or one trial supported by confirmatory evidence, such as a trial for a different infection [22]. Sulopenem/probenecid and gepotidacin trials’ evidence of efficacy was based on subgroup analyses with contradictory results, and inferiority findings for other indications [22]. A prior study also found that imipenem/cilastatin/relebactam, indicated for hospital-acquired bacterial pneumonia and ventilator-associated bacterial pneumonia and cUTI, lacked robust efficacy evidence [15]. These findings suggest the continued need for adequate and well-controlled clinical trials to provide robust evidence of efficacy [14,23].

Efficacy analyses for both antibiotics were conducted post hoc on underpowered, unplanned, non-randomized subgroups, stratifying subjects by active control-susceptible and resistant pathogens, which is an approach that directly conflicts with FDA guidance and standards [22,24,25,26]. In trial 301, sulopenem/probenecid failed to demonstrate noninferiority to ciprofloxacin in subjects with pathogens susceptible to the active control, but it showed superiority in subjects with resistant bacteria [7]. In contrast, in trial 310 sulopenem/probenecid showed noninferiority to amoxicillin/clavulanate in subjects with pathogens susceptible to active control and superiority in subjects with resistant pathogens.

It is worth noting that in trial 301, subjects with *resistant* pathogens who also received concomitant antibacterial therapy were considered as “successes” in the primary efficacy assessment. Had these subjects been excluded from the analysis, sulopenem/probenecid would have shown noninferiority, rather than superiority, compared to ciprofloxacin [7].

Furthermore, in trial 301, subjects with *susceptible* pathogens, who received concomitant antibacterial therapy, were considered as clinical failures based on uUTI symptoms and/or microbiological failures, introducing bias into the sulopenem/probenecid efficacy evaluation. In trial 310, although efficacy data appeared to favor sulopenem/probenecid over the amoxicillin/clavulanate control in the resistant bacteria subgroup, the sample size was too small to make any meaningful inferences [7].

Additionally, analysis of the clinical trials excluded several randomized subjects, in direct contradiction with FDA guidance precluding post-randomization exclusions [22]. Excluding data on micro-ITT populations, including those subjects who withdrew due to adverse events, undermines the reliability of findings. Additionally, several randomized subjects were excluded from micro-ITT analyses, in direct contradiction again with FDA guidance [22].

### 3.2. Clinical Significance of Surrogate Endpoints

All phase III clinical trials of sulopenem/probenecid and gepotidacin used a composite endpoint, clinical cure and microbiological response, as the primary measure of efficacy. *Clinical cure* rates for nitrofurantoin (63–66%) were lower than in previous studies (79–92%) [27,28]. Additionally, previous studies found that untreated patients with uUTI experienced symptom resolution: 42% of patients within a week and over 50% by 35–49 days [29,30]. These findings suggest that the clinical value of new antibiotics for uUTI over available therapies may be overstated in the results from the clinical trials [30].

The FDA guidance recommends that the *test-of-cure (TOC)* assessment after randomization to be timed according to the duration and pharmacokinetics (half-life) of the investigated drug [22]. However, in trial 301, the TOC was performed seven days *after* the end of treatment for sulopenem/probenecid and nine days for ciprofloxacin. In clinical practice, treatment response is typically expected within only a couple of days of starting therapy [31]. Outcomes evaluation should occur at the same time point in both cases and control groups and should not be anchored to post-randomization events such as duration of therapy or end of treatment [22]. Assessing drug efficacy and safety at different time points can bias results, particularly in short, acute illnesses.

Results for clinical and *microbiological response* in sulopenem/probenecid and gepotidacin trials were inconsistent. In gepotidacin trials, many subjects were classified as not achieving therapeutic success, despite showing a successful response in one component of the composite endpoint. The low microbiological success in nitrofurantoin was expected, as per its FDA label nitrofurantoin lacks the broader tissue distribution of other antibiotics approved for UTI [32]. These findings highlight the inherent limitations of relying on pathogen-focused surrogate endpoints to establish efficacy [23,33]. Furthermore, microbiological response provides little insight into clinically meaningful benefits for patients with uUTI, since asymptomatic bacteriuria is not treated in clinical practice and provides no clinical benefit while potentially contributing to antibiotic resistance.

### 3.3. Population Included in Clinical Trials vs. Approved Indication

Sulopenem/probenecid was approved for women with limited or no alternative oral treatment options. However, clinical trials enrolled a broader patient population, and the comparator arms did not include many other available antibiotics. As of July 2025, the FDA had approved 84 antibacterial drugs for UTI, 51 of which are currently marketed, including 10 with oral formulations specifically approved for uUTI [34,35,36].

Per FDA guidance, antibacterial efficacy in noninferiority trials should be assessed in randomized subjects with a pathogen *fully susceptible* to the active control [22,24]. Nevertheless, the sulopenem/probenecid and gepotidacin trials included subjects with pathogens that were either *susceptible or resistant* to the active control, contrary to FDA guidance and internationally accepted standards [22,24,25,26,37]. Active controls used in these trials were not evaluated in subjects with resistant pathogens at the time of their approval. Including subjects with resistant pathogens in the control arm artificially reduces the apparent efficacy of the comparators and introduces bias in favor of the investigational drug, overstating their relative benefit.

### 3.4. Choice of Active Comparators

Sulopenem/probenecid was evaluated against ciprofloxacin and amoxicillin/clavulanate, neither of which are approved for first-line treatment of uUTI [1,38] nor are standard of care [1,39] as recommended by the FDA guidance [22]. Although amoxicillin–clavulanate was approved for uUTIs caused by susceptible organisms, it is rarely used in clinical practice for uncomplicated cystitis and is generally reserved for patients in whom first-line agents, such as nitrofurantoin, trimethoprim–sulfamethoxazole, fosfomycin trometamol, or pivmecillinam, are unsuitable or contraindicated [40,41].

Gepotidacin was evaluated against nitrofurantoin [42], a first-line therapy for uUTI [43]. However, nitrofurantoin’s FDA approval label states that its limited distribution may predispose patients to persistent or recurrent bacteriuria and result in lower eradication rates. The label also cautions that these lower eradication rates should be weighed against the risk of systemic toxicity and antimicrobial resistance posed by broad-spectrum antibiotics [44]. The use of comparators that are neither standard of care nor adequate for comparative analysis raises concerns about the validity of the efficacy assessment and the potential risks to patients.

### 3.5. Safety in Anti-Infective Noninferiority Trials

The incidence of adverse events for both sulopenem/probenecid and gepotidacin was higher than their active controls. Notably, gepotidacin risks the development of antimicrobial resistance and required serious adverse event warnings to be included in its FDA approval label and a medication guide to alert healthcare providers and patients [9,12,45]. The use of noninferiority designs for a mild and self-limited condition, where established therapies are safe and efficacious, raises ethical concerns. The safety profiles of sulopenem/probenecid and gepotidacin in noninferiority trials may conflict with the principle of beneficence, given their worse safety profile than established therapies with comparable efficacy [14,15].

This study has limitations primarily related to reliance on publicly available data. Unpublished trials data may provide additional insights into the efficacy and safety of sulopenem/probenecid and gepotidacin. Our analysis excluded early-phase (phase I and II) trials and nonclinical studies and focused on pivotal phase III trials relevant to regulatory approval.

## 4. Materials and Methods

This study used publicly available regulatory and clinical trials data submitted to the FDA as part of the new drug application (NDA) review process for sulopenem/probenecid and gepotidacin. Study data were derived from the FDA integrated reviews and approval labels available at the Drugs@FDA, the National Library of Medicine DailyMed, and Clinicaltrials.gov. We also reviewed FDA regulations and guidance for the industry and conducted a scoping review of the literature.

We identified eligible clinical trials by cross-referencing FDA reviews with ClinicalTrials.gov registrations. We collected data from trials 301 (NCT03354598) and 310 (NCT05584657) for sulopenem/probenecid. We excluded phase III studies for complicated UTI (Trial 302, NCT03357614) and intra-abdominal infection (Trial 303, NCT03358576) because the FDA did not consider these results supportive of the uUTI indication. We also collected data from Trials EAGLE-2 (NCT04187144) and EAGLE-3 (NCT05630833) for gepotidacin. For both sulopenem/probenecid and gepotidacin, we collected data from pivotal phase III clinical trials. We also reviewed FDA guidance documents to extract recommendations on trials’ design.

Two study investigators performed data abstraction using a pre-defined and piloted data extraction form. In cases of discrepancy, a third investigator reviewed the extracted data, and all discrepancies were resolved by consensus.

We conducted a structured comparative effectiveness assessment to assess clinical trials. Primary endpoints included differences in composite, clinical, and microbiological outcomes, and the proportion of patients experiencing adverse events and treatment discontinuations. We also assessed FDA reviewers’ conclusions regarding trial design, adherence to FDA regulation and guidance, and clinical significance of endpoints reported by the sponsor company. Additionally, we compared the planned clinical trial protocols with the actual conduct of the trials. We assessed patient enrollment criteria, sample size, randomization procedures, comparators, planned versus reported primary and secondary endpoints, timing of outcomes assessment, subgroup analyses reported, and protocol amendments. We conducted a descriptive synthesis given the heterogeneity across trials and regulatory decisions. All reported outcomes, treatment differences between study arms, and corresponding 95% confidence intervals were abstracted directly from FDA review documents. The PICOS elements for each pivotal trial are summarized in Table 5, highlighting key differences in enrolled populations, comparators, and primary outcome definitions.

## 5. Conclusions

This study assessed the application of regulatory standards and FDA guidance in the approval of newest antibiotics for uUTI and found significant flaws in pivotal clinical trial designs that undermine the validity of the trials’ results and limit relevance in clinical practice. Evidence of effectiveness for sulopenem/probenecid and gepotidacin is limited, based on trials conducted in small, non-pre-specified subgroups of subjects with either susceptible *or* resistant pathogens, with contradictory results. These trials did not include standard-of-care antibiotics available in the US market for uUTI and relied on surrogate endpoints assessed *after* a self-limiting condition is largely resolved. Patient-centered outcomes, such as symptom resolution, time to relief, and functional recovery, provide more clinically meaningful evidence to inform regulatory and clinical decision-making than pathogen-focused surrogate endpoints. Higher adverse event rates compared to active controls, combined with the risk for antimicrobial resistance, raise serious safety and ethical concerns.

Future trials for novel uUTI antibiotics should include representative patient populations, standard-of-care comparators, well-controlled study designs, and patient-centered outcomes to provide robust, clinically relevant evidence. Notably, both sulopenem/probenecid and gepotidacin were granted expedited review designations, including Qualified Infectious Disease Product, fast-track, and priority review, despite treating a self-limiting condition with limited evidence of clinical benefit and a safety profile inferior to available therapeutic alternatives.

## Figures and Tables

**Table 1:** Sulopenem etzadroxil/probenecid and gepotidacin phase III clinical trials.

**Sulopenem Etzadroxil/Probenecid Phase 3 Clinical Trials**						
**Trial #NCT Number**	**Start** **Date**	**Completion** **Date**	**Enrollment** **(N)**	**Infection**	**Comparators**	**FDA Review** **Composite Outcomes**
Trial 301 NCT03354598	1 August 2018	20 January 2020	1671	uUTI	Ciprofloxacin	Inferior in control-susceptible pathogens (micro-MITTS). Superior in control-resistant pathogens (micro-MITTR).
Trial 302 NCT03357614	18 September 2018	14 December 2019	1395	cUTI	Ertapenem–ciprofloxacin or amoxicillin–clavulanate	Inferior to controls.
Trial 310 NCT05584657	18 October 2022	21 November 2023	2229	uUTI	Amoxicillin–clavulanate	Superior in control-susceptible pathogens (micro-MITTS). Numerically inferior in control-resistant pathogens (micro-MITTR).
**Gepotidacin Phase III Clinical Trials**						
**Trial #NCT Number**	**Start** **Date**	**Completion** **Date**	**Enrollment (N)**	**Infection**	**Comparators**	**FDA Review** **Composite Outcomes**
NCT04020341	17 October 2019	30 November 2022	1531	uUTI	Nitrofurantoin	Noninferior.
EAGLE-2 NCT04187144	23 April 2020	1 December 2022	1606	uUTI	Nitrofurantoin	Noninferior.
EAGLE-3 NCT06597344	11 January 2023	2 February 2024	380	uUTI	Nitrofurantoin	Superior in active control susceptive bacteria (micro-MITTS).
EAGLE-J NCT05630833	2 October 2024	10 March 2025	97	uUTI	No control	No active control.

NCT: National Clinical Trial number. uUTI: uncomplicated urinary tract infection (UTI). cUTI: complicated UTI. micro-MITTS: Microbiologic modified intent-to-treat subgroup, susceptible to the active control. micro-MITTR: Microbiologic modified intent-to-treat subgroup, resistant to the active control.

**Table 2:** Sulopenem/probenecid phase III clinical trials for uncomplicated UTI.

Trial Number/NCT Number	Trial 301/NCT03354598			Trial 310/NCT05584657		
Outcomes at Test-of-Cure Visit (12 Days After Randomization)	Sulopenem/Probenecid n/N (%)	Ciprofloxacin n/N (%)	Treatment Difference (95% CI)	Sulopenem/Probenecid n/N (%)	Amoxicillin/ Clavulanate n/N (%)	Treatment Difference (95% CI)
**micro-MITTS Population**						
Composite response (primary outcome)	227/376 (60.4)	300/418 (71.8)	−11.4 (−17.9, −4.8) †	296/480 (61.7)	243/442 (55.0)	6.7 (0.3, 13.0) §
Clinical cure	205/376 (81.1)	351/418 (84.0)	−2.9 (−8.2, 2.4)	371/480 (77.3)	339/442 (76.7)	0.6 (−4.8, 6.1)
Microbiological success	262/376 (69.7)	336/418 (80.4)	−10.7 (−16.7, −4.7)	361/480 (75.2)	295/442 (66.7)	8.5 (2.6, 14.3)
**micro-MITTS Results (Composite Response)**	sulopenem/probenecid statistically inferior			sulopenem/probenecid statistically superior		
**micro-MITTR Population**						
Composite response (primary outcome)	78/162 (48.1)	49/149 (32.9)	15.3 (4.3, 25.8) ‡	22/42 (52.4)	17/25 (68.0)	−15.6 (−37.5, 9.1)
Clinical cure	136/162 (84.0)	96/149 (64.4)	19.5 (10.0, 29.0)	26/42 (61.9)	18/25 (72.0)	−10.1 (−31.5, 14.0)
Microbiological success	92/162 (56.8)	66/149 (44.3)	12.5 (1.4, 23.3)	29/42 (69.0)	20/25 (80.0)	−11.0 (−30.7, 12.0)
**micro-MITTR Results (Composite Response)**	sulopenem/probenecid statistically superior			sulopenem/probenecid numerically inferior		

NCT: National Clinical Trial. micro-MITTS: Microbiologic modified intent-to-treat subgroup, susceptible to the active control. micro-MITTR: Microbiologic modified intent-to-treat subgroup, resistant to the active control. Statistically significant differences: § *p* = 0.006; † *p* not available; ‡ *p* = 0.04. Composite responses reflect FDA conclusions rather than statistical outcomes. Susceptible and resistant subgroup analyses were not pre-specified primary analyses.

**Table 3:** Gepotidacin phase III clinical trials for uncomplicated UTI.

Trial Number/NCT Number	EAGLE-2/NCT04187144			EAGLE-3/NCT05630833		
Outcomes at Test-of-Cure Visit (10–13 Days After Randomization)	Gepotidacin n/N (%)	Nitrofurantoin n/N (%)	Treatment Difference (95% CI)	Gepotidacin n/N (%)	Nitrofurantoin n/N (%)	Treatment Difference (95% CI)
**micro-MITTS Population**						
Composite response (primary outcome)	174/336 (51.8)	140/298 (47.0)	5.3 (−2.4, 13.0)	172/292 (58.9)	121/275 (44.0)	14.4 (6.4, 22.4)
Clinical cure	224/336 (66.7)	196/298 (65.8)	1.5 (−5.8, 8.8)	199/292 (68.2)	175/275 (63.6)	4.3 (−3.4, 12.0)
Microbiological success	244/336 (72.6)	199/298 (66.8)	6.0 (−1.2, 13.1)	213/292 (72.9)	158/275 (57.5)	15.5 (7.9, 23.1)
**micro-MITTS Results (Composite Response)**	*gepotidacin noninferior and numerically superior*			*gepotidacin superior*		
**micro-MITTR Population**						
Composite response (primary outcome)	24/65 (36.9)	17/67 (25.4)	10.7% (−4.7, 26.1)	27/39 (69.2)	15/49 (30.6)	33.9 (16.4, 51.5)
Clinical cure	30/65 (46.2)	34/67 (50.7)	NA	32/39 (82.1)	26/49 (53.1)	NA
Microbiological success	50/65 (76.9)	38/67 (56.7)	17.9 (2.8, 33.0)	31/39 (79.5)	24/49 (49.0)	24.7 (6.8, 42.6)
**micro-MITTS Results** **(Composite Response)**	*gepotidacin noninferior and numerically superior*			*gepotidacin superior*		

NA: Not available. micro-MITTS: Microbiologic modified intent-to-treat subgroup, susceptible to the active control. micro-MITTR: Microbiologic modified intent-to-treat subgroup, resistant to the active control. Composite outcomes reflect FDA conclusions rather than statistical outcomes. n = subgroup analysis sample size and N = total sample size.

**Table 4:** Sulopenem and gepotidacin pooled FDA safety analyses treatment-emergent adverse events.

	Sulopenem Active Comparators			Gepotidacin Active Comparator	
Adverse Event (AE)	Sulopenem (N = 1932)	Amoxicillin/Clavulanate (N = 1107)	Ciprofloxacin (N = 822)	Gepotidacin (N = 1570)	Nitrofurantoin (N = 1558)
Any AE	416 (21.5%)	136 (12.3%)	115 (14.0%)	551 (35.1%)	365 (23.4%)
Serious AEs	6 (0.3%)	5 (0.5%)	2 (0.2%)	24 (1.5%)	14 (0.9%)
AE leading to treatment discontinuation	21 (1.1%)	4 (0.4%)	8 (1.0%)	79 (5.0%)	30 (1.9%)

**Table 5:** PICOS framework to strengthen evidence gathered in clinical trials.

PICOS Element	Trial 301	Trial 310	EAGLE-2	EAGLE-3
**P.** Population	Eligibility: Adult women ≥18 years with acute uUTI (24–96 h of symptoms), ≥2 symptoms: dysuria, urgency, frequency, suprapubic pain, baseline urine culture ≥10^5^ CFU/mL of *Enterobacterales* or *S. saprophyticus*, required dipstick or pyuria confirmation. Exclusions: pyelonephritis signs, antibiotics within prior 7 days, urinary tract abnormalities, immunocompromised, pregnancy/lactation, significant renal/hepatic disease, other.	Same population characteristics and eligibility criteria as Trial 301, with minor administrative differences (e.g., explicit exclusions for valproic acid use, gout, history of seizures). Same clinical inclusion criteria and uUTI definition.	Eligibility: Nonpregnant females ≥12 years, ≥40 kg, presenting within 96 h of onset of signs/symptoms of acute cystitis (dysuria, urgency, frequency, suprapubic pain), required pyuria or nitrite positivity. Exclusions: immunocompromised, anatomical abnormalities, renal impairment (CrCl < 60 mL/min), QT prolongation risk, prior antimicrobial therapy, acute cystitis known or suspected, symptoms of complicated UTI, pregnancy, pathogens not susceptible to nitrofurantoin, other.	Same inclusion/exclusion criteria as EAGLE-2. Population characteristics highly similar (mean age ~48 years, majority non-Hispanic White, ~60 percent nonrecurrent infections).
**I.** Intervention	Sulopenem etzadroxil 500 mg + probenecid 500 mg PO twice daily for 5 days.	Same intervention as trial 301.	Gepotidacin 1500 mg PO twice daily for 5 days (10 doses total).	Same regimen as EAGLE-2.
**C.** Comparator	Ciprofloxacin 250 mg PO twice daily for 3 days.	Amoxicillin/clavulanate 875/125 mg PO twice daily for 5 days.	Nitrofurantoin 100 mg PO twice daily for 5 days (10 doses total).	Same comparator as EAGLE-2.
**O.** Outcomes (Primary and Key Secondary)	Primary: Composite overall success (clinical cure + microbiologic eradication) at Test-of-Cure (TOC; Day 12 (±1 days)). Separate analyses for ciprofloxacin-susceptible (Micro-MITTS) and ciprofloxacin-resistant (Micro-MITTR) pathogens. Secondary: clinical cure, microbiologic eradication, subgroup analyses.	Same as in Trial 301. Primary and secondary endpoints use the same clinical and microbiologic criteria, but susceptibility groups are defined using amoxicillin/clavulanate (Micro-MITTS, Micro-MITTR).	Primary: Composite response at TOC; days 10–13 in Micro-ITT nitrofurantoin-susceptible isolates (NTF-S) population. Composite success required: (1) microbiological eradication of baseline pathogens (<10^3^ CFU/mL) and (2) complete clinical symptom resolution. Secondary: Clinical response at TOC; days 10–13 and follow-up (FU), microbiologic response at TOC and FU, sustained composite response at FU.	Same primary and secondary endpoints as EAGLE-2.
**S.** Study design	Phase 3, multicenter, randomized, double-blind, double-dummy, active-controlled NI trial.	Design is the same as trial 301: Phase 3, randomized, double-blind, double-dummy, active-controlled NI trial.	Multicenter, double-blind, double-dummy, randomized (1:1), parallel-group, noninferiority design; conducted across 107 sites in 12 countries. NI margin: 10 percent. Includes PK sampling.	Nearly identical design as in EAGLE 2; conducted across 112 sites in 6 countries. Included post-treatment ECG monitoring but no PK sampling.

NTF-S: nitrofurantoin-susceptible isolates; NI: Noninferiority. Test-of-cure (TOC); PO (*per os*) by mouth. micro-MITTS: Microbiologic modified intent-to-treat subgroup, susceptible to the active control. micro-MITTR: Microbiologic modified intent-to-treat subgroup, resistant to the active control.

## Data Availability

Data are contained within the article. The data used for the study are available at the FDA website Drugs@FDA.
